# Supplementary material for: Effect of a NICU to Home Physical Therapy Intervention on White Matter Trajectories, Motor Skills, and Problem-Solving Skills of Infants Born Very Preterm: A Case Series
Source: J Pers Med. 2022 Dec 7;12(12):2024. doi: 10.3390/jpm12122024 (PMC9784100; doi:10.3390/jpm12122024)
Supplement: Supplementary file 1 [file jpm-12-02024-s001.zip › Figure S1.pdf]

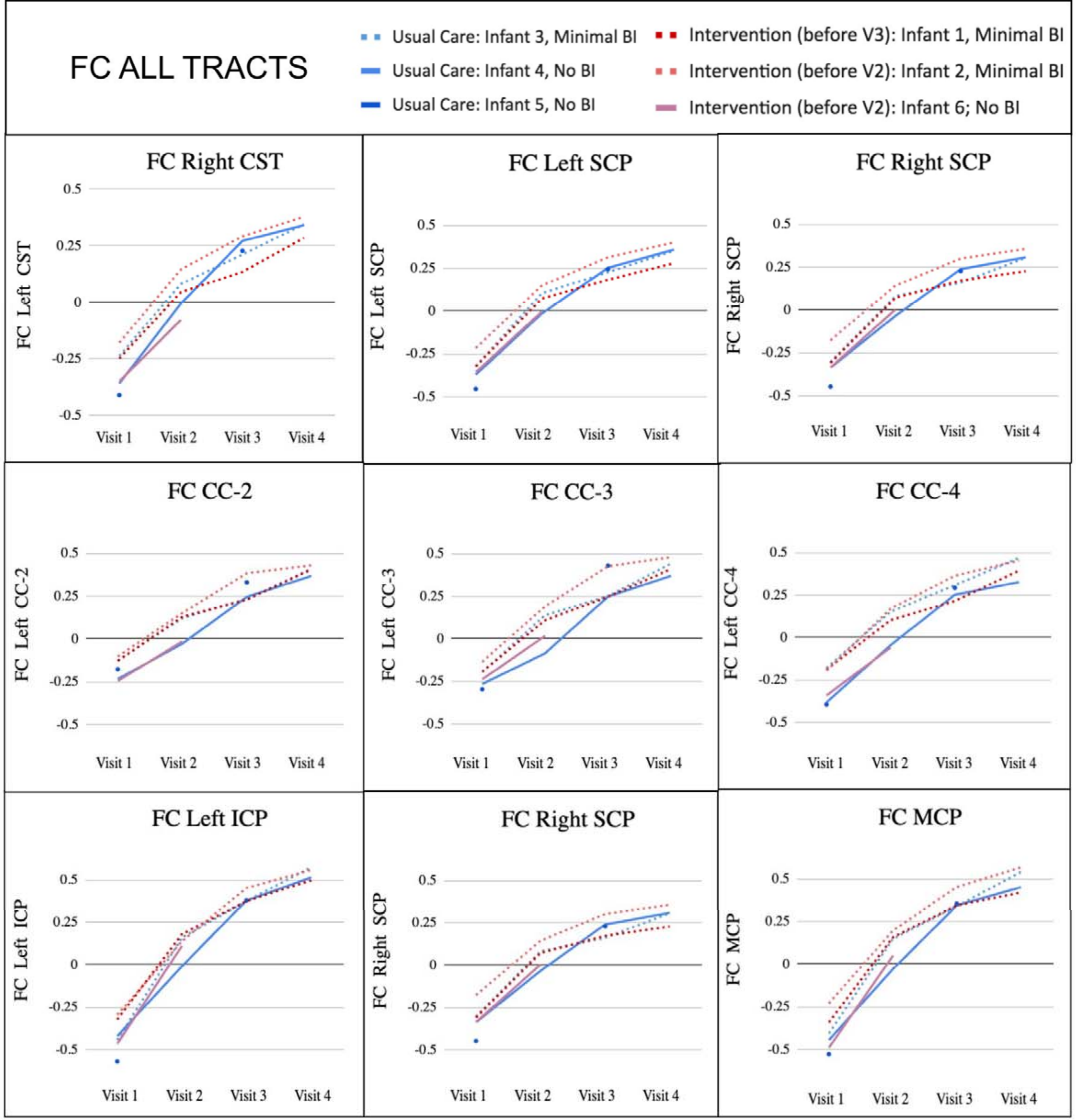
FC ALL TRACTS
Usual Care: Infant 3, Minimal BI
Usual Care: Infant 4, No BI
Usual Care: Infant 5, No BI
Intervention (before V3): Infant 1, Minimal BI
Intervention (before V2): Infant 2, Minimal BI
Intervention (before V2): Infant 6; No BI
FC Right CST
FC Left CST
FC Left SCP
FC Left SCP
FC Right SCP
FC Right SCP
FC CC-2
FC Left CC-2
FC CC-3
FC Left CC-3
FC CC-4
FC Left CC-4
FC Left ICP
FC Left ICP
FC Right SCP
FC Right SCP
FC MCP
FC MCP
0.5
0.25
0
-0.25
-0.5
Visit 1
Visit 2
Visit 3
Visit 4
