# Supplementary material for: Effect of a NICU to Home Physical Therapy Intervention on White Matter Trajectories, Motor Skills, and Problem-Solving Skills of Infants Born Very Preterm: A Case Series
Source: J Pers Med. 2022 Dec 7;12(12):2024. doi: 10.3390/jpm12122024 (PMC9784100; doi:10.3390/jpm12122024)
Supplement: Supplementary file 1 [file jpm-12-02024-s001.zip › Figure S2.pdf]

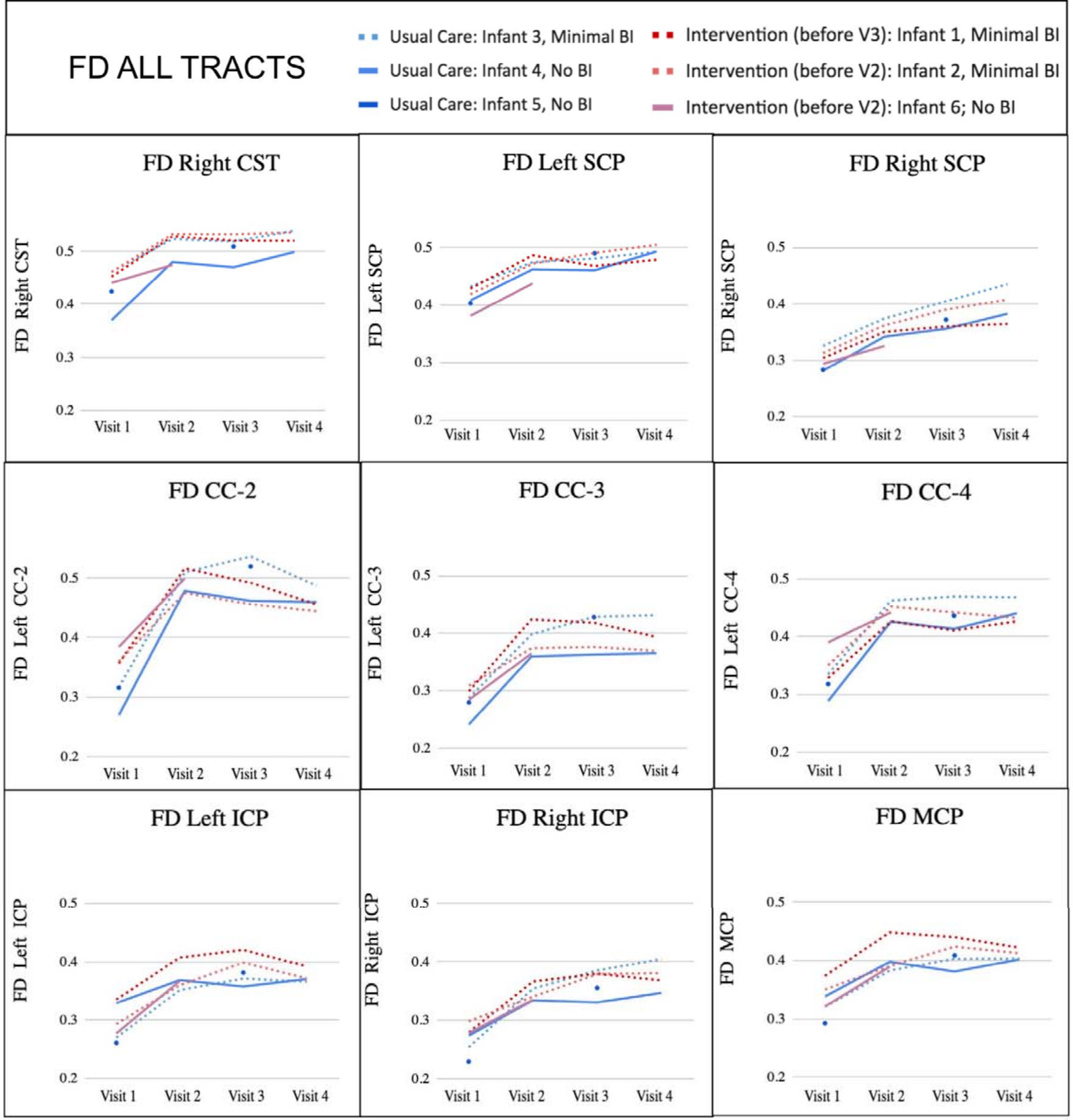
FD ALL TRACTS
Usual Care: Infant 3, Minimal BI
Usual Care: Infant 4, No BI
Usual Care: Infant 5, No BI
Intervention (before V3): Infant 1, Minimal BI
Intervention (before V2): Infant 2, Minimal BI
Intervention (before V2): Infant 6; No BI
FD Right CST
FD Right CST
FD Left SCP
FD Left SCP
FD Right SCP
FD Right SCP
FD CC-2
FD Left CC-2
FD CC-3
FD Left CC-3
FD CC-4
FD Left CC-4
FD Left ICP
FD Left ICP
FD Right ICP
FD Right ICP
FD MCP
FD MCP
0.2
0.3
0.4
0.5
Visit 1
Visit 2
Visit 3
Visit 4
