# Supplementary material for: Effect of a NICU to Home Physical Therapy Intervention on White Matter Trajectories, Motor Skills, and Problem-Solving Skills of Infants Born Very Preterm: A Case Series
Source: J Pers Med. 2022 Dec 7;12(12):2024. doi: 10.3390/jpm12122024 (PMC9784100; doi:10.3390/jpm12122024)
Supplement: Supplementary file 1 [file jpm-12-02024-s001.zip › Figure S3.pdf]

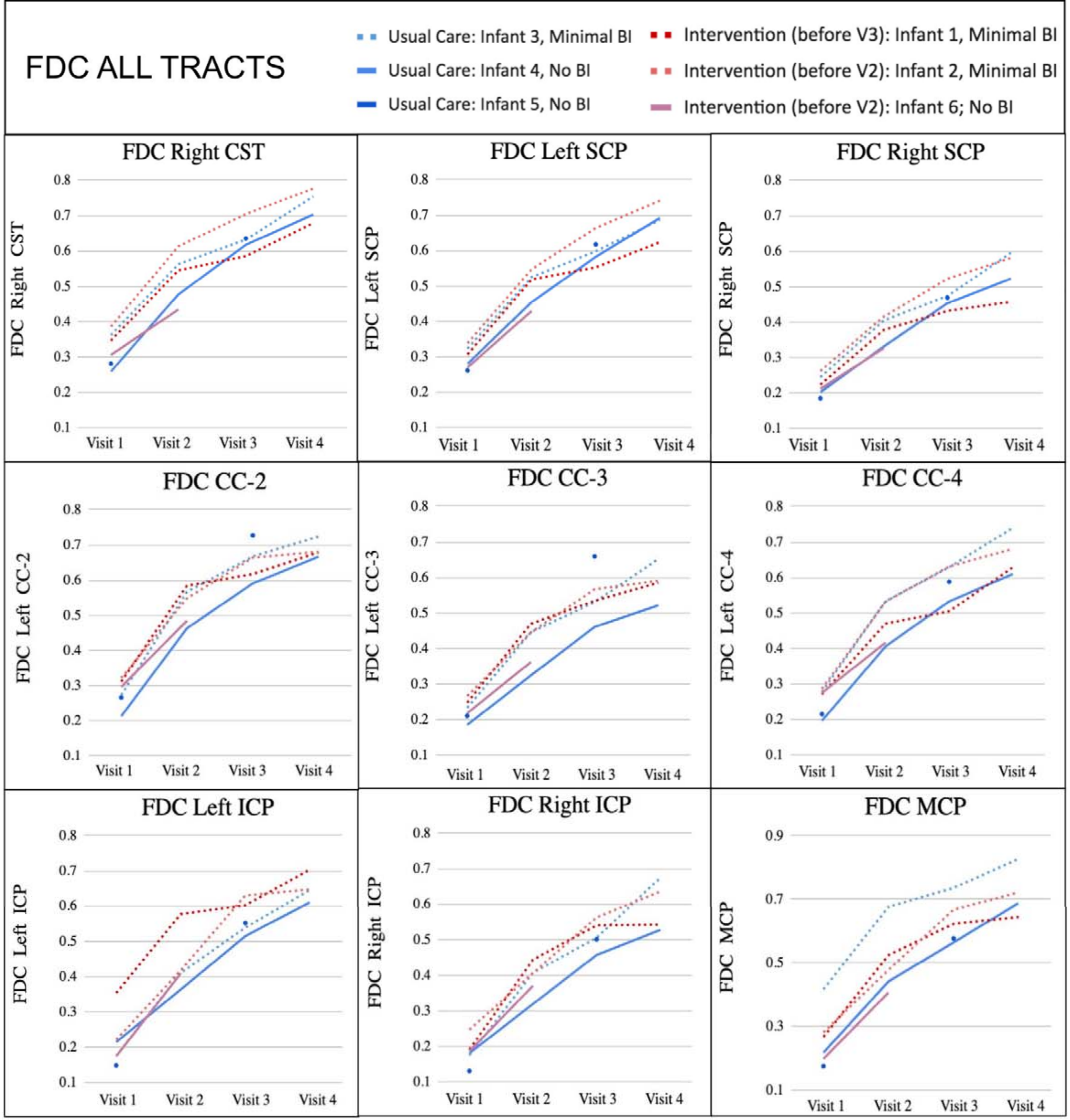
FDC ALL TRACTS
Usual Care: Infant 3, Minimal BI
Usual Care: Infant 4, No BI
Usual Care: Infant 5, No BI
Intervention (before V3): Infant 1, Minimal BI
Intervention (before V2): Infant 2, Minimal BI
Intervention (before V2): Infant 6; No BI
FDC Right CST
FDC Left SCP
FDC Right SCP
FDC CC-2
FDC CC-3
FDC CC-4
FDC Left ICP
FDC Right ICP
FDC MCP
FDC Right CST
FDC Left SCP
FDC Right SCP
FDC Left CC-2
FDC Left CC-3
FDC Left CC-4
FDC Left ICP
FDC Right ICP
FDC MCP
Visit 1
Visit 2
Visit 3
Visit 4
